# Radiometrically Equivalent Comparison of 254 nm Low-Pressure Mercury Lamps and InGaN 280 nm UV–C LEDs for Surface Disinfection

**DOI:** 10.1021/acsomega.6c07484

**Published:** 2026-09-18

**Authors:** Janaise Mesquita Bueno Furtado, Everton Granemann Souza, Chiara das Dores do Nascimento, Carolina Bacchieri Falcão, Marcel Luiz Basso, Mario Lucio Moreira

**Affiliations:** † Graduate Program in Physics (PPGFis), 37902 Federal University of Pelotas (UFPel), Pelotas, Rio Grande do Sul 96010-900, Brazil; ‡ Inovare BioSim Lab, 67714 Catholic University of Pelotas (UCPel), Pelotas, Rio Grande do Sul 96015-560, Brazil; § Graduate Program in Electronics and Computer Engineering (MEEC), 564099 Catholic University of Pelotas (UCPel), Pelotas, Rio Grande do Sul 96015-560, Brazil; ∥ Graduate Program in Materials Science and Engineering (PPGCEM), Federal University of Pelotas (UFPel), Pelotas, Rio Grande do Sul 96010-610, Brazil; ⊥ Graduate Program in Sciences and Technologies in Education (PPGCITED), Federal Institute Sul-rio-grandense (IFSul), Pelotas, Rio Grande do Sul 96060-290, Brazil; # Graduate Program in Electronic and Computer Engineering (MEEC), Catholic University of Pelotas (UCPel), Pelotas, Rio Grande do Sul 96015-560, Brazil

## Abstract

Ultraviolet germicidal irradiation (UVGI) is traditionally based on low-pressure mercury lamps emitting at 254 nm, whereas UV–C light-emitting diodes (LEDs) have emerged as promising mercury-free alternatives. However, direct comparisons between these technologies are frequently confounded by differences in irradiance, exposure geometry, and accumulated UV–C dose, making it difficult to isolate wavelength-dependent effects from dosimetric bias. In this study, two closed irradiation chambers equipped with a low-pressure Hg lamp (254 nm) and an InGaN UV–C LED array (280 nm) were comprehensively evaluated in terms of emission spectrum, temporal irradiance stability, and spatial dose distribution. Radiometric measurements established matched irradiation conditions corresponding to source-to-sample distances of 41.0 cm for the Hg lamp and 5.5 cm for the LED system. For microbiological testing, both sources were operated for 270 s to reach stable irradiance, while the plates remained shielded from UV–C until the timed exposure began. Under matched accumulated UV–C doses, *Staphylococcus aureus* ATCC 25923 exhibited comparable microbial inactivation kinetics. A single-exponential decay model with a residual offset yielded similar model-estimated characteristic doses (D_90_, D_99_, and D_99.9_), while the survivor curves agreed in order of magnitude with historical and contemporary susceptibility data. Pairwise comparisons identified a statistically significant difference at only one exposure condition, whereas ANCOVA did not detect a statistically significant dose × technology interaction. These findings demonstrate that, when rigorous radiometric equivalence is established, the apparent performance differences between 254 and 280 nm become substantially smaller than generally inferred from nominal wavelength alone.

## Graphical abstract

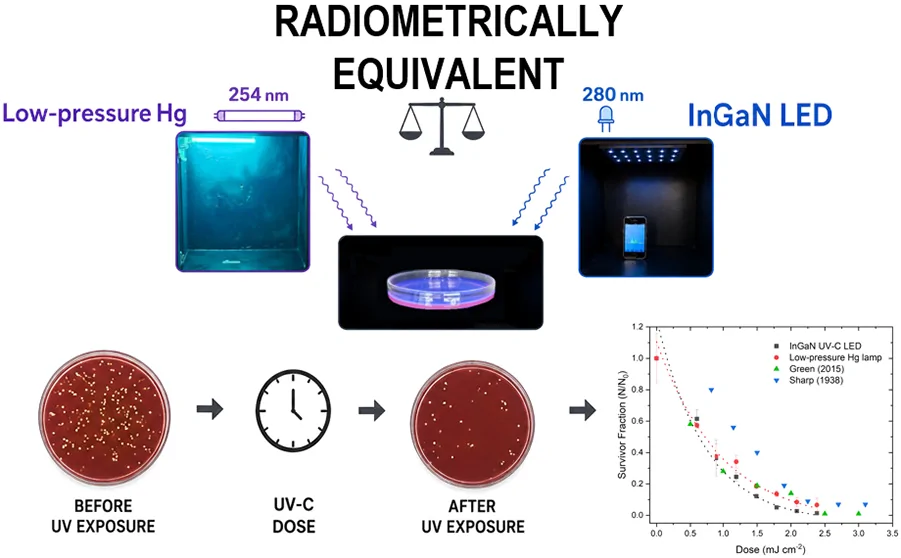

## Introduction

Ultraviolet germicidal irradiation (UVGI), particularly within the UV–C spectrum (200–280 nm), has evolved into one of the most effective nonchemical technologies for microbial control due to its rapid germicidal activity, residue-free operation, and compatibility with automated disinfection systems. Initially established for drinking water treatment, UVGI is now widely employed for potable and wastewater disinfection,^1^ healthcare environmental decontamination,^2–5^ food processing and preservation,^6,7^ laboratory biosafety cabinets and containment-related decontamination procedures,^8^ medical device sterilization,^9^ and UV-based advanced oxidation processes (UV-AOPs) for the degradation of emerging contaminants and wastewater treatment.^10^ This remarkable expansion has been driven by the growing demand for sustainable, rapid, and chemical-independent technologies capable of mitigating microbial transmission in increasingly complex environments, establishing UVGI as a strategic engineering platform at the interface of environmental, industrial, and biomedical sciences.

For more than five decades, low-pressure mercury lamps emitting predominantly at 253.7 nm have served as the benchmark for germicidal irradiation.^11,12^ Their widespread adoption results from the high absorption of nucleic acids near this wavelength, promoting the formation of cyclobutane pyrimidine dimers and other DNA photoproducts that inhibit replication and cellular division.^13,14^ Consequently, mercury lamps established the experimental basis of classical UV susceptibility studies and continue to underpin engineering design methodologies and microbial dose–response databases. Nevertheless, increasing environmental restrictions associated with mercury-containing devices,^15^ together with limitations related to warm-up time, fragility, fixed emission spectrum, and restricted optical flexibility, have stimulated the search for alternative UV–C technologies.^16^

Among these alternatives, semiconductor-based UV–C light-emitting diodes (UV–C LEDs) have emerged as one of the most promising innovations in photonic disinfection. Compared with conventional mercury lamps, UV–C LEDs offer mercury-free operation, compact dimensions, instantaneous switching, wavelength selectivity, low-voltage operation, and greater flexibility for optical design and system integration.^4,17^ Continuous advances in AlGaN-based deep-UV emitters, together with recent progress in InGaN-based UV emitters operating near the UV–C range, have substantially increased optical output power, wall-plug efficiency, and operational lifetime, enabling applications ranging from decentralized water treatment and portable sterilization devices to autonomous healthcare robots and smart disinfection platforms.^18,19^

However, replacing mercury lamps with UV–C LEDs is not merely a change of light source, since microbial inactivation depends on wavelength, spectral bandwidth, irradiance distribution, accumulated dose, exposure geometry, and the physiological state of the microorganism.^20^ Although nucleic acids absorb strongly near 260 nm, other cellular targets, including proteins, membrane-associated chromophores, and enzymes, also absorb within the UV–C region.^21^ Therefore, nearby wavelengths such as 254 and 280 nm may promote different relative contributions of DNA damage, protein photochemistry, membrane alteration, oxidative stress, and impairment of repair pathways.^14^ For this reason, germicidal performance cannot be predicted solely from nucleic-acid absorption spectra, and meaningful comparisons between mercury lamps and UV–C LEDs require controlled radiometric conditions.

Although several studies have compared UV–C LEDs with low-pressure mercury lamps for water disinfection, analogous investigations focused on air and surface disinfection systems remain comparatively limited.^4,22,23^ In these systems, replacing a mercury lamp with a UV–C LED often modifies not only the emission wavelength, but also the irradiance field, beam profile, source-to-sample distance, optical configuration, and accumulated dose, making it difficult to distinguish intrinsic wavelength-dependent biological effects from confounds introduced by the experimental setup.

Addressing this limitation requires comparative studies in which UV–C sources are spectrally and radiometrically characterized, exposure conditions are rigorously matched, and microbial survival is analyzed using quantitative dose–response metrics that can be directly benchmarked against both historical susceptibility models and contemporary experimental data.

The present work tests the hypothesis that, when low-pressure Hg lamps (254 nm) and InGaN UV–C LEDs (280 nm) are evaluated under rigorously equivalent accumulated UV–C doses established through complete radiometric characterization, their microbial inactivation kinetics become substantially more similar than generally inferred from nominal wavelength alone. To test this hypothesis, two closed irradiation chambers were designed and experimentally characterized with respect to emission spectra, temporal irradiance stability, and spatial dose distribution, providing the dosimetric foundation needed to minimize radiometric bias. Microbiological exposures were initiated only after a 270-s source-stabilization period, with the plates shielded from UV–C until the beginning of the timed exposure. Subsequently, *Staphylococcus aureus* ATCC 25923 was exposed to matched accumulated UV–C doses delivered by both systems, and the resulting dose–response curves were analyzed using a single-exponential decay model with a residual offset and compared with both classical susceptibility data and recent literature.

## Experimental Section

### UV–C Irradiation Systems

#### UV–C Irradiation Chamber at 254 nm

The 254 nm UV–C irradiation chamber was constructed from six MDF panels assembled into a closed enclosure with internal dimensions of 55 × 40 × 47 cm, resulting in an approximate internal volume of 0.1034 m^3^, as shown in Figure 1a. The internal surfaces were coated with a matte black finish to minimize UV–C reflections and reduce the contribution of secondary radiation originating from the chamber walls.

**Figure 1:**
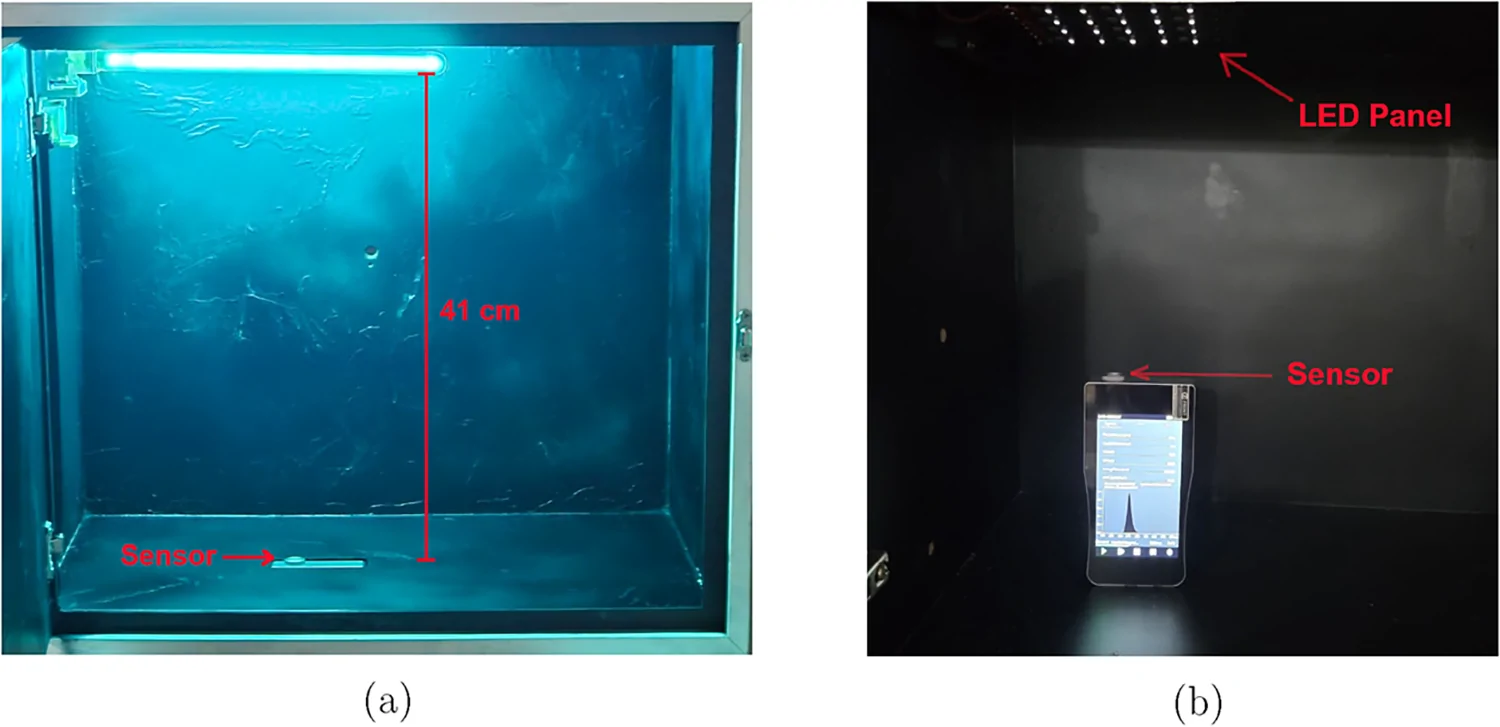
Experimental UV–C irradiation systems used in this study. (a) Irradiation chamber equipped with a low-pressure germicidal mercury lamp (254 nm); (b) irradiation chamber equipped with UV–C LED modules (280 nm), showing the geometric configuration employed for UV–C irradiance and dose measurements, including the detector positioning relative to the radiation source.

The radiation source consisted of a low-pressure germicidal mercury lamp (OSRAM PURITEC HNS L 24 W 2G11, OSRAM GmbH, Munich, Germany), with a nominal electrical power of 24 W, a specified radiant output of 8.5 W in the 200–280 nm range, and peak emission at 254 nm. These manufacturer-rated values correspond to a lamp-level electrical-to–UV-C conversion efficiency of approximately 35.4% (8.5/24 × 100).^24^ The lamp was mounted horizontally near the midplane of the chamber, allowing direct irradiation of microbiological samples positioned at the bottom of the system.

#### UV–C Irradiation Chamber at 280 nm

The 280 nm UV–C irradiation chamber employed the same mechanical structure and internal geometry as the 254 nm chamber (Figure 1b), differing only in the radiation source.

The UV–C source was assembled from four 5-W linear LED modules (SumTang, Shenzhen Santang Lighting Co., Ltd., Shenzhen, China) mounted parallel and side-by-side without intentional gaps. Each module measured 145 × 20 × 2 mm and contained five manufacturer-specified InGaN-based UV–C emitters operating in the 265–280 nm range. The complete source therefore comprised 20 emitters arranged in a 4 × 5 matrix, with a nominal electrical power of 20 W and an overall panel footprint of approximately 145 × 80 mm. The approximate center-to-center spacings were 22.5 mm between emitters along each module and 20 mm between adjacent modules, giving an emitter-center span of approximately 90 × 60 mm.

The manufacturer specified a radiant output of 40–70 mW per module and a nominal full viewing angle of 120°. The four-module array therefore had a manufacturer-rated UV–C output of 0.16–0.28 W, corresponding to a nominal electrical-to–UV-C conversion efficiency of approximately 0.8–1.4% based on its 20-W rated electrical power. At the working distance of 5.5 cm, the nominal viewing angle corresponds to a geometrical beam diameter of approximately 19.1 cm for each emitter.

The array was powered by an external DC power supply operating at 15.0 V and 1.26 A, corresponding to a measured DC electrical input of 18.9 W under the experimental conditions. The microbiological plates were centered beneath the array on an elevated sample holder, with the agar surface positioned 5.5 cm below the emitting plane. Because the emission fields of the individual LEDs overlapped, the effective exposure was established from the irradiance measured directly at the sample plane rather than inferred from the nominal viewing angle or electrical power. In both chambers, exposure time was controlled using a programmable smart switch (Sonoff DIY TH10, Shenzhen Sonoff Technologies Co., Ltd., Shenzhen, China).

### Spectral Characterization

The emission spectra of the UV–C sources were obtained using a UV–vis spectrometer (Flex STD, Sarspec, Porto, Portugal) coupled to a 200 μm optical fiber. The fiber tip was positioned along the principal emission axis at 25 cm from the 254 nm UV–C source and at 5.5 cm from the 280 nm UV–C source. These distances were selected to obtain comparable signal intensities, with maximum counts close to 80,000 in both spectra, avoiding detector saturation. Spectral acquisition was performed under steady-state operating conditions using Light Scan software (version 1.1.17), with an integration time of 2 ms. After acquisition, the spectra were normalized to a 0–1 scale to allow direct comparison of the emission profiles.

### Radiometric Characterization

The spatial distribution of UV–C radiation inside the chambers was characterized through irradiance measurements performed with a UV spectroradiometer (LMS-6000UV, Lisun Group, Shanghai, China). Measurements were conducted along the central irradiation axis, corresponding to the position subsequently adopted in the microbiological assays. All radiometric measurements were performed under controlled laboratory conditions, with ambient temperature of approximately 20 °C and relative humidity of approximately 50%.

For both chambers, measurements were initiated after a warm-up period of 4 min and 30 s to ensure stabilization of the UV–C emission. After this period, the sources remained continuously energized throughout the entire acquisition sequence, avoiding fluctuations associated with repeated switching cycles.

The spectroradiometer sensor was successively repositioned at different source-to-detector distances while maintaining all other experimental conditions unchanged. Eleven distances were evaluated for each chamber, ranging from 1 to 41 cm for the 254 nm system and from 1 to 23 cm for the 280 nm system. At each position, the detector was oriented directly toward the emitting source, maintaining normal incidence relative to the irradiated plane.

The accumulated UV–C dose was estimated from the average irradiance measured at each distance, considering a fixed exposure time of 12 s, selected as a reference interval close to the maximum exposure time used in the subsequent microbiological assays. The dose was calculated according to

1 D=E×t

where *D* is the accumulated UV–C dose (mJ cm^–2^), *E* is the irradiance (mW cm^–2^), and *t* is the exposure time (s).

### Microorganism and Inoculum Preparation

Microbiological assays were performed using *S. aureus* ATCC 25923 (Plastlabor, Rio de Janeiro, RJ, Brazil). The strain was initially reactivated in Brain Heart Infusion broth (BHI; Kasvi, São José dos Pinhais, PR, Brazil) at 37 °C for 24 h. An aliquot of the broth culture was then streaked onto Mannitol Salt Agar (Kasvi, São José dos Pinhais, PR, Brazil) and incubated at 37 °C for an additional 24 h to obtain isolated colonies.

A well-isolated colony was aseptically transferred into sterile distilled water and dispersed by vortex mixing. The turbidity of the resulting suspension was adjusted to a 0.5 McFarland standard, nominally corresponding to approximately 1.5 × 10^8^ CFU mL^–1^, using a Plus II turbidimeter (Alfakit, Florianópolis, SC, Brazil).

Following standardization, serial 10-fold dilutions were prepared from this suspension in sterile distilled water. The 10^–5^ dilution was selected for the UV–C irradiation assays because it provided a suitable concentration for colony enumeration and was used consistently throughout the experiments.

### Microbiological Assays

For the microbiological assays, 100 μL aliquots of the 10^–5^ dilution prepared from the standardized *S. aureus* suspension were uniformly spread onto Mannitol Salt Agar plates.

Three nonirradiated control plates and three replicate plates were prepared for each irradiation condition. The experiments were performed using the UV–C irradiation chambers described in the UV–C Irradiation Systems subsection, subsection, equipped with a low-pressure Hg lamp (254 nm) and an InGaN LED array (280 nm). The plates were positioned directly beneath the radiation sources. For the Hg-lamp system, the plates were placed on the chamber floor, with the agar surface positioned 41 cm below the lamp. For the LED system, the plates were placed on an elevated sample holder, with the agar surface positioned 5.5 cm below the emitting plane of the LED array.

These distances were established from the radiometric characterization presented in the Dose as a Function of Distance subsection to provide equivalent irradiance at the sample plane. This procedure compensated for the different radiant powers, emission geometries, and spatial irradiance distributions of the two technologies, allowing a direct microbiological comparison under identical exposure times and accumulated UV–C doses.

Exposure times ranged from 2 to 8 s. Following irradiation, the plates were incubated at 37 °C for 24 h, after which bacterial survival was quantified by colony-forming unit (CFU) enumeration. The results were expressed as mean CFU per plate, percentage reduction relative to the nonirradiated control, and logarithmic reduction.

Before each microbiological exposure, the irradiation source was operated for 270 s while the plate remained at the predefined sample position and was protected by a UV–C-opaque barrier. After source stabilization, the barrier was removed to initiate the timed exposure. At the end of the programmed interval, the irradiation source was automatically deactivated using the smart switch. This procedure prevented preirradiation of the samples and ensured that the 2–8 s exposure intervals were delivered under steady-state irradiance conditions.

The microbiological performance of the two UV–C technologies was assessed using complementary statistical approaches. First, mean CFU values obtained at each exposure time/dose were compared using Welch’s *t*-test, which is appropriate for small independent samples and does not assume equal variances between groups. To control the family wise error rate associated with multiple pairwise comparisons, the resulting *p*-values were adjusted using the Bonferroni correction, adopting an overall significance level of α = 0.05.

Second, the complete dose–response data sets were analyzed by analysis of covariance (ANCOVA), using log_10_(CFU) as the dependent variable, accumulated UV–C dose as a continuous covariate, irradiation technology (254 nm Hg lamp or 280 nm InGaN LED) as a categorical factor, and the dose × technology interaction term. The ANCOVA was considered the primary statistical analysis because it evaluates the entire dose–response relationship rather than individual dose levels. The significance of the interaction term was used to determine whether the relationship between accumulated UV–C dose and microbial survival depended on the irradiation technology. A nonsignificant interaction indicates that no statistically significant evidence was found that the dose–response relationship differed between the two irradiation technologies over the investigated dose range.

All statistical analyses were performed using GraphPad Prism 10 (GraphPad Software, San Diego, CA), and differences were considered statistically significant at *p* < 0.05.

## Results and Discussion

### Spectral Characterization of the UV–C Sources

The spectral emission profiles of the UV–C sources used in this study are presented in Figure 2. Distinct spectral characteristics were observed for the low-pressure mercury lamp and the UV–C LED system, reflecting the fundamentally different physical mechanisms responsible for photon generation in each technology.

**Figure 2:**
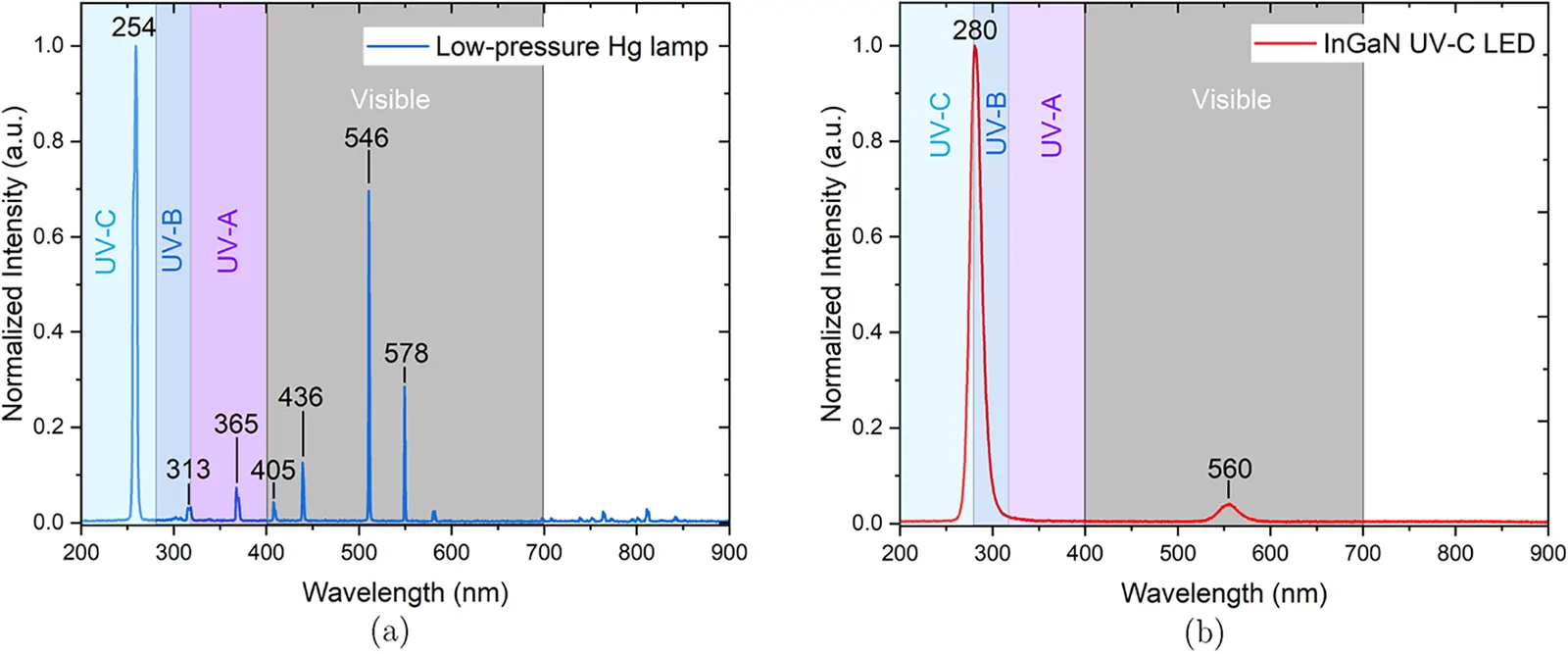
Normalized emission spectra of the UV–C sources used in this study: (a) low-pressure mercury germicidal lamp and (b) InGaN-based UV–C LED. The mercury lamp exhibits a dominant narrow emission line at 254 nm, accompanied by secondary Hg emission lines at approximately 313, 365, 405, 436, 546, and 578 nm. In contrast, the UV–C LED shows a broader emission band centered at approximately 280 nm, together with a weak broad visible band centered near 560 nm. The shaded areas indicate the corresponding spectral regions.

The low-pressure mercury lamp (Figure 2a) exhibits an intense and narrow emission peak centered at approximately 254 nm, corresponding to the principal resonance line of atomic mercury. This emission lies within the germicidal UV–C region and accounts for most of the radiant power emitted by the source. In addition to the dominant peak, several characteristic mercury emission lines are observed at approximately 313, 365, 405, 436, 546, and 578 nm. These secondary emissions originate from electronic transitions between excited states of mercury atoms within the discharge plasma and constitute the characteristic spectral fingerprint of low-pressure mercury germicidal lamps. The simultaneous presence of these lines confirms the gas-discharge nature of the source and agrees with the emission pattern commonly reported for mercury-vapor UV lamps.

In contrast, the UV–C LED source (Figure 2b) exhibits a broad emission band centered at 280.8 nm with a full width at half-maximum (FWHM) of approximately 15 nm. Unlike the mercury lamp, whose emission is concentrated in discrete atomic lines, the LED spectrum arises from radiative electron–hole recombination processes within the semiconductor active region. Such broad-band emission is characteristic of nitride-based UV–C LEDs and results from the energetic distribution of charge carriers as well as compositional and structural inhomogeneities within the active layers of the device. The measured FWHM is consistent with values commonly reported for commercial UV–C LEDs and reflects the intrinsic spectral broadening associated with carrier recombination processes in semiconductor heterostructures.^18,25^

A weak broad emission band is also observed in the visible region, centered near 560 nm. Although its intensity is substantially lower than that of the main UV–C peak, similar visible emissions have been reported in GaN- and InGaN-based devices and are commonly attributed to defect-related luminescence involving deep electronic states, impurities, and local compositional fluctuations within the semiconductor structure.^26^

The spectral differences between both UV–C technologies are particularly relevant for microbial inactivation applications. While the mercury lamp concentrates most of its radiant power in an extremely narrow spectral line at 254 nm, close to the maximum absorption region of nucleic acids, the LED distributes its emission over a broader wavelength interval centered near 280 nm. Because the absorption spectra of biological chromophores are wavelength dependent, variations in spectral distribution may influence the relative contribution of photochemical damage mechanisms involving nucleic acids, proteins, and other cellular constituents. In particular, nucleic acids exhibit maximum absorption near 260 nm, whereas proteins containing aromatic amino acids absorb more strongly at slightly longer wavelengths. Consequently, radiation sources operating at different UV–C wavelengths may promote distinct balances between DNA damage and protein photodegradation during microbial inactivation.^20^

Overall, the results highlight the fundamental distinction between gas-discharge UV–C systems, characterized by quasi-monochromatic atomic emission lines, and semiconductor-based UV–C systems, characterized by broader emission bands centered at specific wavelengths. These differences are expected to influence the photochemical interactions between UV radiation and microbial targets and may contribute to variations in disinfection performance observed under equivalent UV doses.

### Temporal Stability of UV–C Irradiance

The temporal evolution of UV–C irradiance measured at the sample plane is presented in Figure 3. Distinct stabilization behaviors are observed for the low-pressure mercury lamp and the InGaN-based UV–C LED, reflecting the different physical mechanisms responsible for radiation generation in each technology.

**Figure 3:**
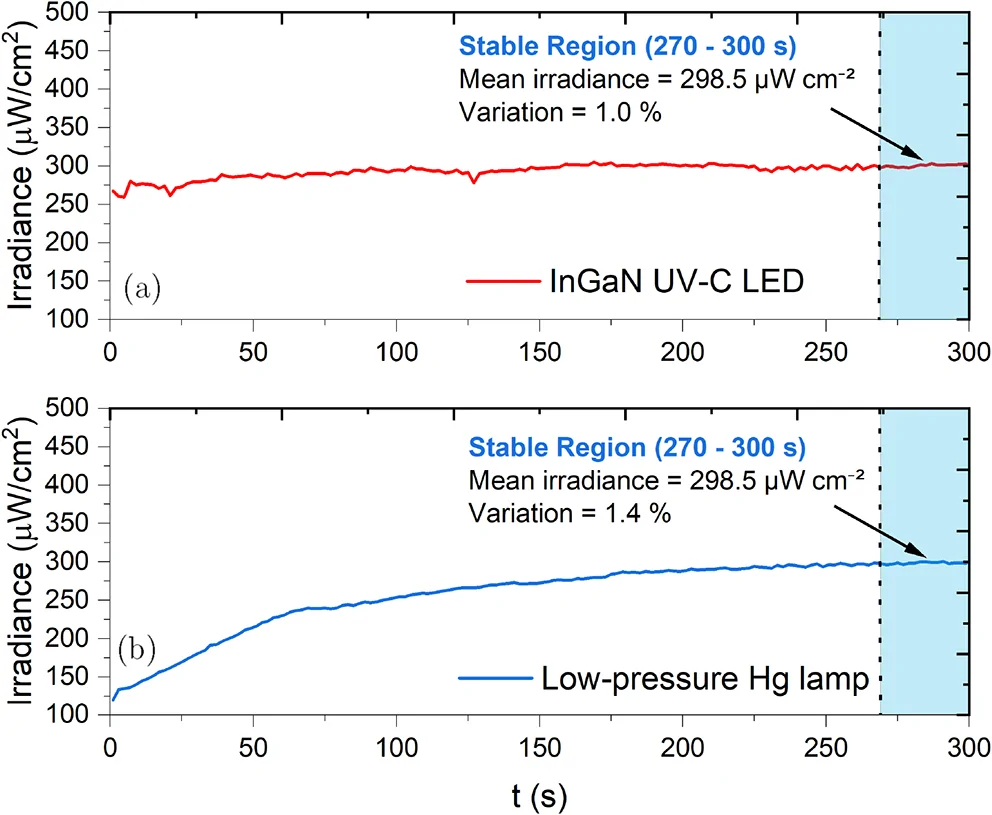
Temporal evolution of UV–C irradiance measured at the sample plane for the irradiation systems employed in this study. (a) InGaN-based UV–C LED operating at a source-to-sample distance of 5.5 cm and (b) low-pressure mercury germicidal lamp operating at a source-to-sample distance of 41 cm. The shaded region corresponds to the stabilization interval (270–300 s) adopted for subsequent radiometric characterization and microbiological experiments.

The UV–C LED source exhibits a nearly constant irradiance throughout the monitoring period. The irradiance increases from approximately 267 μW cm^–2^ immediately after activation to values close to 299 μW cm^–2^ at the end of the measurement, corresponding to an overall variation of approximately 12.0%. Such behavior is characteristic of semiconductor-based UV emitters, in which photon generation results from direct electron–hole recombination within the active region of the device. Consequently, UV–C LEDs reach their operating condition almost instantaneously and do not require the warm-up period typically associated with discharge-based lamps.^25,27^

In contrast, the low-pressure mercury lamp exhibits a pronounced increase in irradiance during the first minutes of operation. The irradiance rises from approximately 120 μW cm^–2^ immediately after activation to approximately 299 μW cm^–2^ after 300 s, corresponding to an increase of approximately 149%. This behavior is attributed to the progressive establishment of thermal equilibrium within the discharge tube. As the lamp temperature increases, mercury vapor pressure approaches its optimum operating condition, resulting in a gradual increase in the intensity of the 254 nm resonance line. Similar warm-up behavior has been extensively reported for low-pressure mercury UV systems and is considered an intrinsic characteristic of gas-discharge lamps used in germicidal applications.^1^

A particularly relevant result is observed within the stabilization interval adopted in this work (270–300 s). In this region, both systems converge to essentially identical average irradiance values of 298.5 μW cm^–2^, despite their markedly different temporal behaviors. The mercury lamp exhibits fluctuations of approximately 1.4%, whereas the UV–C LED shows a variation of approximately 1.0%, indicating stable operating conditions for both technologies during the experimental period.

To achieve this condition, the source-to-sample distance was adjusted independently for each irradiation system. A distance of 41 cm was adopted for the low-pressure mercury lamp, whereas a distance of 5.5 cm was used for the UV–C LED source. These distances were selected based on the radiometric characterization presented in the following section, allowing both systems to deliver the same average irradiance at the sample plane. This approach minimizes radiometric bias during microbiological comparisons and ensures that differences in microbial inactivation can be attributed primarily to spectral characteristics and source technology rather than differences in irradiance.

The convergence of both systems to the same irradiance level is particularly important because UV dose is directly proportional to irradiance and exposure time. Consequently, maintaining equivalent irradiance conditions allows a more rigorous comparison between the 254 nm mercury lamp and the 280 nm LED source. This strategy is especially relevant when comparing mercury-based and semiconductor-based UV–C technologies, which differ substantially in emission mechanism, spectral distribution, thermal behavior, and operational characteristics.^25,27^

Another important observation is the excellent temporal stability exhibited by both systems after stabilization. Once thermal equilibrium is reached, irradiance fluctuations remain below 1.5% for both sources, demonstrating a high degree of radiometric reproducibility. Such stability is desirable in UV disinfection studies because it reduces uncertainty in dose delivery and improves the reliability of dose–response relationships obtained during microbial inactivation experiments.

Overall, the results demonstrate that both irradiation systems provide stable and reproducible UV–C output after a stabilization period of 270 s. However, the markedly different start-up behaviors highlight an important operational advantage of semiconductor-based UV–C systems. While the UV–C LED reaches near-steady-state operation immediately after activation, the low-pressure mercury lamp requires several minutes to achieve its nominal irradiance. Although both technologies ultimately deliver equivalent irradiance under the conditions employed in this study, the rapid stabilization of UV–C LEDs represents a practical advantage for applications requiring frequent switching, intermittent operation, and precise control of delivered UV dose.^27^

### Dose as a Function of Distance

The accumulated UV–C dose as a function of source-to-sample distance is presented in Figure 4 for both irradiation systems using a 12 s exposure time, selected as a reference interval close to the maximum exposure time used in the subsequent microbiological assays. Measurements were performed within the 270–300 s interval after chamber activation, previously identified as the stable operating window, to ensure that the reported dose values reflected the steady-state emission of each source.

**Figure 4:**
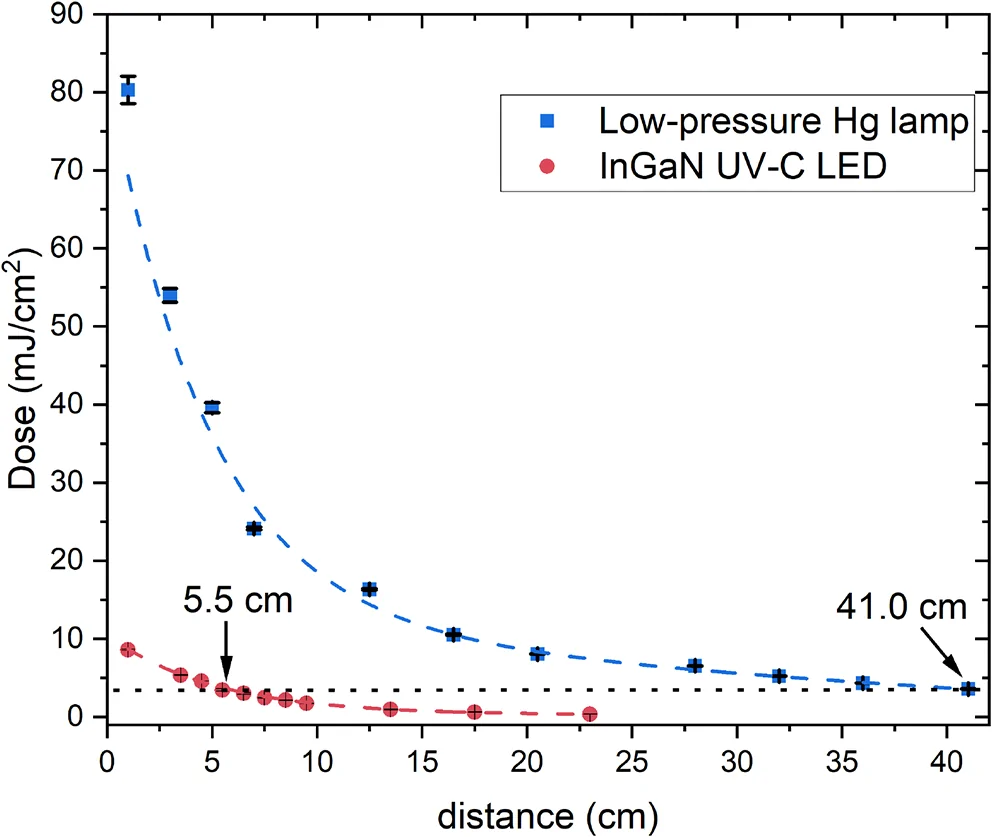
Accumulated UV–C dose as a function of source-to-sample distance for the low-pressure mercury lamp (254 nm) and the InGaN UV–C LED source (280 nm) using an exposure time of 12 s. Symbols represent the experimental mean values and dashed lines correspond to empirical exponential fits (*R* ^2^ = 0.983 for the Hg lamp and *R* ^2^ = 0.999 for the UV–C LED). The horizontal dotted line indicates the equivalent dose condition adopted for the microbiological assays. The arrows identify the selected irradiation distances of 41.0 cm for the mercury lamp and 5.5 cm for the UV–C LED.

For both technologies, the delivered dose decreases monotonically with increasing distance from the radiation source. However, substantial differences are observed in both the magnitude of the dose and the spatial attenuation profile. The low-pressure mercury lamp produces considerably higher doses at short distances, reaching approximately 80 mJ cm^–2^ at 1 cm, whereas the UV–C LED source delivers approximately 9 mJ cm^–2^ under the same condition. This difference reflects the distinct emission characteristics of the two technologies, including source geometry, radiant flux, and spatial power distribution.

The experimental data were well described by a second-order exponential decay function

2 $$D\left(d\right)=y_{0}+A_{1}e^{-d/t_{1}}+A_{2}e^{-d/t_{2}}$$

where *D*(*d*) is the accumulated UV–C dose and *d* is the source-to-sample distance. The fitted curves yielded coefficients of determination of *R* ^2^ = 0.983 for the 254 nm mercury lamp and *R* ^2^ = 0.999 for the 280 nm UV–C LED source, indicating that the dose attenuation within the investigated range was primarily governed by the source-to-sample distance. The corresponding fitting parameters are summarized in Table 1.

**Table 1:** Fitting Parameters for the Accumulated UV–C Dose as a Function of Source-to-Sample Distance Using a Second-Order Exponential Decay Model^a^

source	*y*_0_	*A*_1_	*t*_1_	*A*_2_	*t*_2_	*R* ^2^
low-pressure Hg lamp	–8.15	95.73	4.81	20.98	72.67	0.983
InGaN UV–C LED	0.250	5.18	5.11	5.12	5.11	0.999

a *D*, *y*_0_, *A*_1_, and *A*_2_ are expressed in mJ cm^–2^, while *d*, *t*_1_, and *t*_2_ are expressed in cm.

Although both systems exhibit a pronounced reduction in dose with increasing distance, the mercury lamp maintains measurable germicidal doses over substantially larger source-to-sample distances. For example, the accumulated dose decreases from approximately 80 to 4 mJ cm^–2^ between 1 and 41 cm, whereas the UV–C LED dose decreases from approximately 9 to values below 1 mJ cm^–2^ over a considerably shorter distance range. This behavior is consistent with the elongated geometry of the mercury discharge tube, which behaves as an extended linear source, whereas the LED array produces a more localized radiation field with a steeper spatial dose gradient.^1,25^

A particularly important result is the identification of equivalent irradiation conditions between the two technologies. As indicated by the horizontal dotted line in Figure 4, a dose of approximately 4 mJ cm^–2^ is obtained at a distance of 41.0 cm for the mercury lamp and 5.5 cm for the UV–C LED source. These distances were therefore selected for all subsequent microbiological experiments.

The establishment of equivalent dose conditions is fundamental for a meaningful comparison between the two UV–C technologies. Since UV dose is one of the primary parameters governing microbial inactivation, matching the delivered dose minimizes radiometric bias and enables the influence of wavelength and source technology to be evaluated more directly. Consequently, differences observed in the microbial response can be more confidently attributed to the distinct spectral characteristics of the 254 nm mercury lamp and the 280 nm UV–C LED source rather than to differences in the amount of UV energy delivered to the samples.

Overall, the results demonstrate that substantially different source-to-sample distances are required to achieve equivalent UV–C doses when comparing mercury-based and semiconductor-based irradiation systems. Nevertheless, the dose-matching strategy adopted in this work provides a robust experimental framework for investigating the influence of UV–C wavelength on microbial inactivation under controlled radiometric conditions.

### Microbial Inactivation and Dose–Response Behavior

Representative culture plates obtained after irradiation with the two UV–C systems are presented in Figure 5, while the corresponding quantitative microbiological results obtained from triplicate experiments at each irradiation condition are summarized in Table 2. Regardless of the irradiation source, increasing the accumulated UV–C dose produced a progressive reduction in the number of viable *S. aureus* colonies, demonstrating a clear dose-dependent microbial inactivation response.

**Figure 5:**
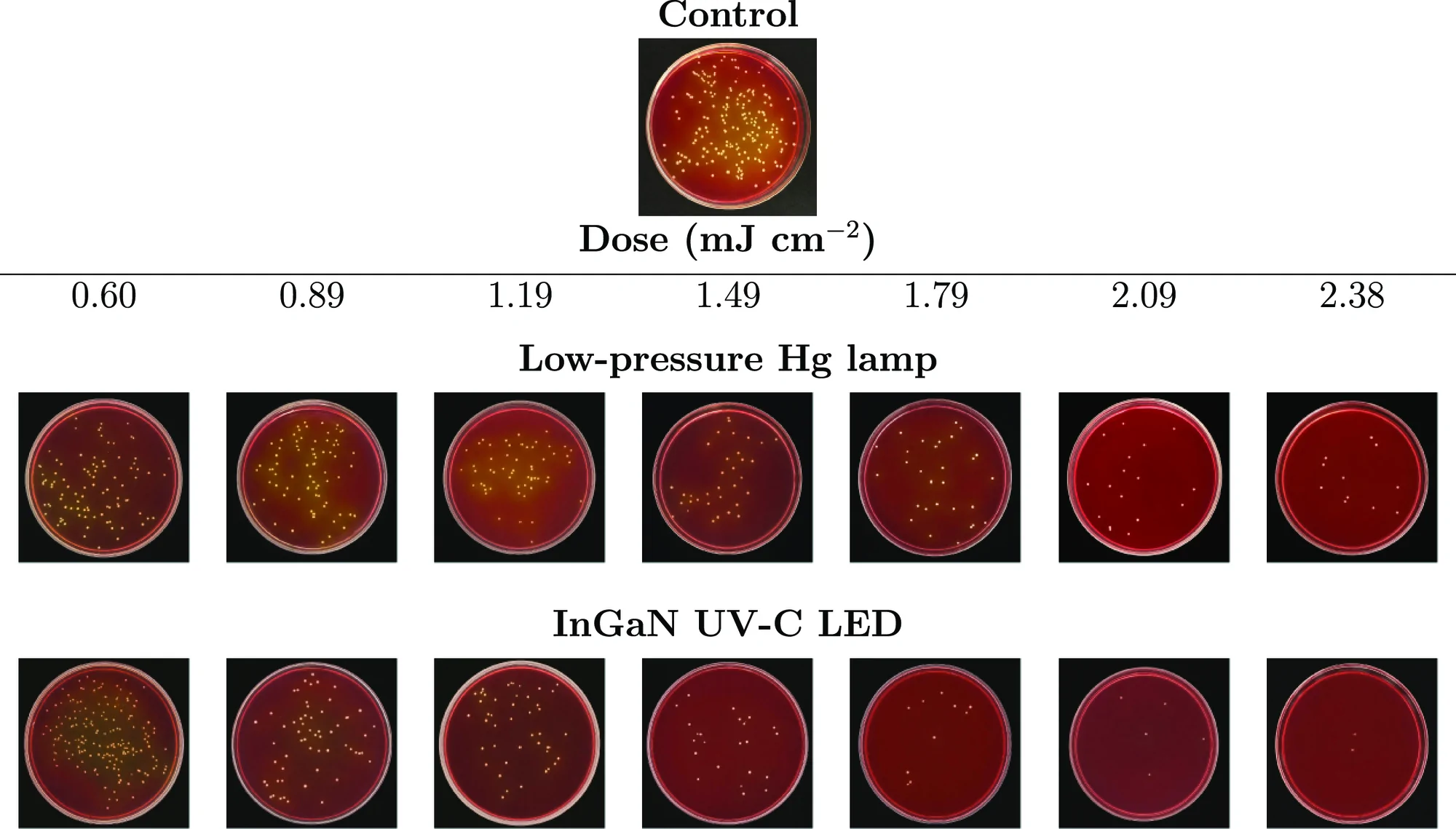
Representative culture plates after UV–C exposure. The control is shown at the top, followed by plates irradiated at 254 nm (low-pressure Hg lamp) and at 280 nm (InGaN UV–C LED). One representative plate is shown for each condition.

**Table 2:** Comparison of the Microbial Inactivation of *S. aureus* Achieved with the Low-Pressure Hg Lamp and InGaN LED Systems under Equivalent Accumulated UV–C Doses And Exposure Times^a^

UV–C source	dose	time	mean ± SD	log_10_	disinfection
	(mJ cm^– 2^)	(s)	(CFU)	reduction	(%)
control	–	–	168.3 ± 27.0	–	0.0
InGaN LED	0.60	2	105.3 ± 10.2	0.20	37.4
	0.89	3	62.7 ± 19.8	0.43	62.7
	1.19	4	42.0 ± 4.6	0.60	75.0
	1.49	5	21.0 ± 1.7^b^	0.90	87.5
	1.79	6	8.0 ± 1.2	1.32	95.2
	2.09	7	4.7 ± 1.5	1.55	97.2
	2.38	8	2.3 ± 1.5	1.86	98.6
low-pressure Hg lamp	0.60	2	98.3 ± 2.1	0.23	41.6
	0.89	3	64.3 ± 13.4	0.42	61.8
	1.19	4	58.7 ± 7.0	0.46	65.1
	1.49	5	31.7 ± 2.1^b^	0.73	81.2
	1.79	6	23.3 ± 3.5	0.86	86.2
	2.09	7	14.7 ± 4.9	1.06	91.3
	2.38	8	12.3 ± 7.5	1.14	92.7

a Mean colony counts (± SD), log_10_ reduction, and disinfection efficiency are reported as a function of UV–C dose.

b Significant difference between UV–C sources at the same exposure time according to Welch’s *t* test with Bonferroni adjustment (*p* < 0.0071).

The quantitative data confirm the visual trends observed in the representative plates. For the InGaN UV–C LED, the average viable count decreased from 168.3 ± 27.0 CFU in the control group to 2.3 ± 1.5 CFU after an accumulated dose of 2.38 mJ cm^–2^, corresponding to a 1.86-log reduction and a disinfection efficiency of 98.6%. Under the same accumulated UV–C dose and exposure time, the low-pressure Hg lamp reduced the bacterial population to 12.3 ± 7.5 CFU, corresponding to a 1.14-log reduction and a disinfection efficiency of 92.7%.

An important observation is that substantial microbial inactivation was achieved at relatively low accumulated doses for both irradiation systems. At 1.49 mJ cm^–2^, the viable bacterial population was reduced by 87.5% with the InGaN UV–C LED and by 81.2% with the low-pressure Hg lamp. At 2.09 mJ cm^–2^, these reductions increased to 97.2 and 91.3%, respectively. Although the UV–C LED produced lower mean CFU counts than the Hg lamp at most accumulated doses, the differences were generally small relative to the experimental variability.

Consistent with this overall overlap, pairwise Welch’s *t*-tests with Bonferroni adjustment identified a statistically significant difference between the two irradiation technologies only at 1.49 mJ cm^–2^, at which the UV–C LED produced greater inactivation. At all other accumulated doses, no statistically significant differences were detected (*p*_adj_ > 0.05), indicating that the data provided no evidence of source-dependent differences under matched-dose conditions.

This conclusion is further supported by the ANCOVA, which showed that the interaction between accumulated UV–C dose and irradiation technology was not statistically significant. Therefore, no evidence was found that the dose–response relationship differed between the low-pressure Hg lamp and the InGaN UV–C LED over the investigated dose range. These statistical results indicate that, when equivalent accumulated UV–C doses are delivered under controlled radiometric conditions, microbial inactivation is primarily governed by the accumulated UV–C dose, whereas the choice of irradiation technology (254 nm Hg lamp or 280 nm InGaN LED) did not produce statistically distinguishable dose–response behavior under the controlled experimental conditions adopted in this study.

This observation deserves particular attention because conventional germicidal UV–C systems are traditionally based on low-pressure mercury lamps emitting predominantly at 254 nm, a wavelength located close to the maximum absorption peak of nucleic acids. Consequently, 254 nm irradiation has historically been regarded as the optimum wavelength for microbial inactivation.^11,28,29^ However, recent investigations have demonstrated that the germicidal effectiveness of UV–C radiation cannot be attributed exclusively to DNA absorption. Wavelengths near 280 nm also interact efficiently with proteins and other intracellular components, while differences in source characteristics, emission bandwidth, irradiance distribution and irradiation geometry may substantially influence the overall inactivation process.^11,30^ As a result, direct statements regarding the superiority of one UV–C wavelength over another should be made cautiously, particularly when the wavelengths are separated by only a few tens of nanometers and equivalent accumulated doses are delivered.

Recent comparative studies have reached similar conclusions, indicating that microbial susceptibility within the germicidal UV–C region depends not only on wavelength, but also on the microorganism, experimental matrix, irradiation geometry and dosimetric methodology.^30^ Consequently, comparisons based exclusively on wavelength may lead to misleading conclusions if the delivered UV–C dose is not rigorously controlled. The present experimental design minimizes these variables by exposing both irradiation systems to identical accumulated doses, allowing a more direct comparison of their intrinsic disinfection performance.

To evaluate the inactivation kinetics across the full dose range, Figure 6 presents the survivor fraction as a function of accumulated UV–C dose for both irradiation sources, together with the data reported by Green et al.^31^ and Sharp^32^ as contemporary and historical benchmarks, respectively.

**Figure 6:**
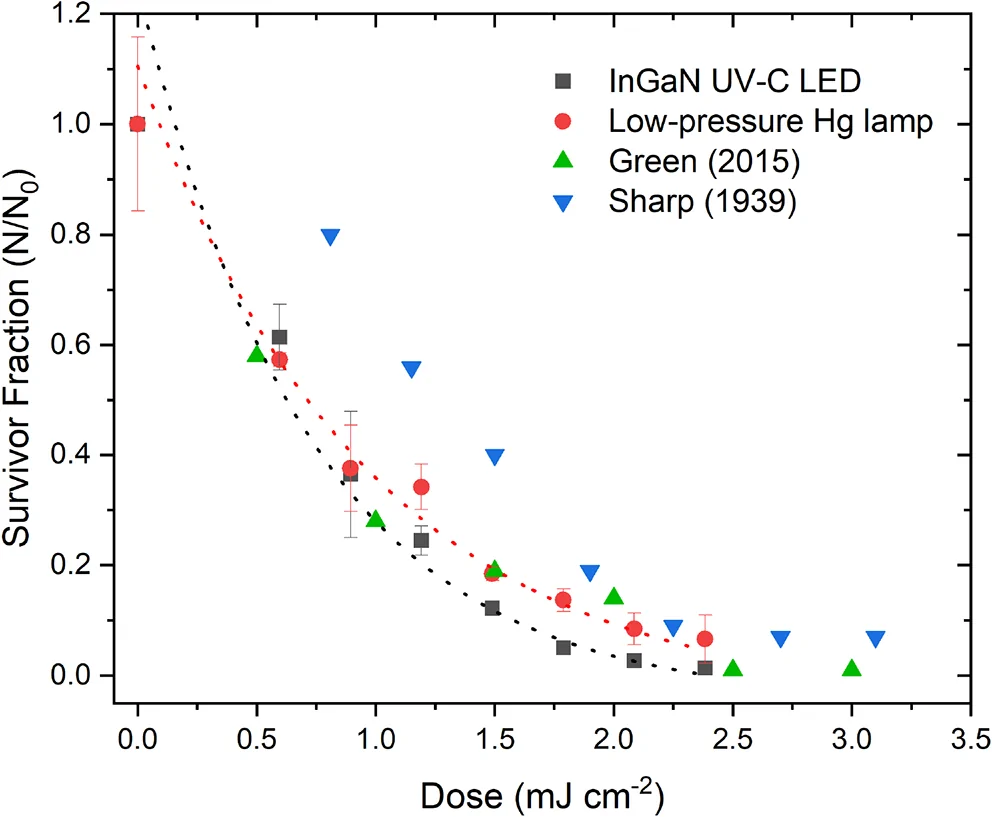
Survivor fraction (*N*/*N*_0_) as a function of accumulated UV–C dose for the 254 and 280 nm irradiation systems used in this work, compared with literature curves reported by Green et al. (2015) and Sharp (1939). Error bars are shown for the experimental data obtained in this study. Standard deviations for the Green et al.^31^ and Sharp^32^ data sets are not shown because these values were not reported in the original publications.

Although the three studies used a comparable experimental framework, their inoculum-preparation protocols differed. Green et al.^31^ cultivated *S. aureus* ATCC 25923 in approximately 100 mL of tryptic soy broth for 24 h at 35 °C under agitation. Following serial dilution, 0.01 mL aliquots were spread onto tryptic soy agar and allowed to dry for 30 min before irradiation. Sharp^32^ also used a 24-h broth culture, which was diluted in normal saline to approximately 1.5 × 10^4^ organisms mL^–1^. A 5 mL volume was distributed over each 9 cm beef-extract agar plate for 20 s, followed by inversion for approximately 40 s to drain the excess liquid, resulting in 600–1000 colonies per plate. Thus, both literature studies used liquid-grown cells that were subsequently deposited and irradiated on agar surfaces. In contrast, the suspension used in the present study was prepared from freshly isolated colonies collected after 24 h of growth on Mannitol Salt Agar. Because neither Green et al. nor Sharp experimentally verified the bacterial growth phase through a growth curve or phase-specific harvesting procedure, their inocula are more appropriately compared in terms of culture age and growth format rather than assigned categorically to the logarithmic or stationary phase.

Despite these methodological differences, the curves obtained with the low-pressure Hg lamp and the InGaN UV–C LED exhibited similar exponential decay trends across the investigated dose range and were broadly consistent with the data reported by Green et al.^31^ and Sharp.^32^ This agreement indicates that both irradiation sources produced the expected dose-dependent inactivation of *S. aureus*, although differences in inoculum preparation preclude strict quantitative equivalence among the data sets.

To quantitatively compare the inactivation kinetics, the experimental data were fitted using a single-exponential decay model with a residual offset, according to eq 3. The exponential component is consistent with the first-order behavior commonly used in UV-disinfection analyses, whereas the adjustable parameters *A*_1_ and *y*_0_ accommodate deviations from an ideal normalized response. Exponential UV-inactivation behavior was described in the classical work of Sharp^32^ and remains widely used in modern UV-disinfection analyses.^11^

3 $$\frac{N}{N_{0}}=A_{1}e^{-D/t_{1}}+y_{0}$$

where *N*/*N*_0_ is the survivor fraction, *D* is the accumulated UV–C dose, *A*_1_ is the pre-exponential coefficient, *t*_1_ is the characteristic dose of the exponential component, and *y*_0_ is the residual offset. The apparent inactivation constant was calculated as *k* = 1/*t*_1_.

The fitted parameters are summarized in Table 3. The data sets were well described by the single-exponential model, with coefficients of determination ranging from 0.950 to 0.995. These results support an approximately exponential dose–response relationship over the investigated interval. Because the fitted values of *y*_0_ were slightly negative, they should be interpreted only as numerical offsets resulting from the unconstrained fitting procedure and not as physically meaningful asymptotic survivor fractions.

**Table 3:** Fitted Parameters Obtained from the Single-Exponential Inactivation Model and Corresponding Model-Estimated Characteristic UV–C Doses^a^

source	*y*_0_	*A*_1_	*t*_1_	*k*	D_90_	D_99_	D_99.9_	*R* ^2^
InGaN UV–C LED	–0.0473	1.299	0.725	1.379	1.58	2.26	2.39	0.950
low-pressure Hg lamp	–0.0515	1.157	0.963	1.038	1.96	2.83	2.98	0.995
Green et al. (2015)	–0.0243	1.022	0.917	1.090	1.93	3.11	3.39	0.976
Sharp (1939)	–0.0274	2.105	0.884	1.132	2.48	3.56	3.80	0.987

a The parameters *y*_0_ and *A*_1_ are dimensionless; *t*_1_, D_90_, D_99_, and D_99.9_ are expressed in mJ cm^–2^; and *k* is expressed in cm^2^ mJ^–1^.

The characteristic dose, *t*_1_, provides a convenient quantitative measure of microbial UV susceptibility because it represents the dose required for the exponential component of the survivor fraction to decrease by a factor of *e*. Consequently, lower values of *t*_1_ and higher values of the inactivation constant, *k*, indicate more rapid microbial inactivation. Although the InGaN UV–C LED exhibited a slightly lower characteristic dose (*t*_1_ = 0.725 mJ cm^–2^) and a correspondingly higher inactivation constant (*k* = 1.38 cm^2^ mJ^–1^) than the low-pressure Hg lamp (*t*_1_ = 0.963 mJ cm^–2^ and *k* = 1.04 cm^2^ mJ^–1^), these differences remained relatively modest and should be interpreted in the context of the overall similarity of the fitted dose–response curves. Likewise, the estimated characteristic doses required to achieve 90, 99 and 99.9% microbial inactivation differed by no more than 0.6 mJ cm^–2^ between both irradiation systems, reinforcing that comparable accumulated UV–C doses are required to obtain equivalent disinfection levels.

Rather than demonstrating a substantial advantage of one irradiation technology over the other, the fitted parameters indicate that both UV–C sources produced remarkably similar inactivation kinetics when evaluated under identical dosimetric conditions. This observation is particularly relevant because comparisons between mercury lamps and UV–C LEDs frequently emphasize wavelength differences without fully accounting for dose equivalence. The present results indicate that, once accumulated UV–C dose is rigorously controlled, the differences between 254 and 280 nm become considerably smaller than often suggested in the literature.

The fitted literature data sets provide useful contextual benchmarks for the present results. The characteristic doses obtained here were close to those derived from Green et al.,^31^ whereas the Sharp data set^32^ yielded slightly higher values. Considering the differences in strain, culture format, physiological condition, growth medium, plate preparation, irradiation geometry, and radiometric procedure, these comparisons establish agreement in the order of magnitude of UV–C susceptibility rather than direct validation across equivalent protocols.

An important implication of the present results concerns the interpretation of wavelength effects in UV–C disinfection. Although 254 nm lies close to the maximum DNA absorption peak and has historically been considered the reference wavelength for germicidal applications, microbial inactivation is a considerably more complex process involving multiple intracellular targets. Recent investigations have demonstrated that wavelengths around 280 nm also produce efficient damage to proteins and enzymatic systems, while the final germicidal response depends simultaneously on the spectral characteristics of the source, irradiance distribution, optical design, dose delivery and the microbial action spectrum. Consequently, direct claims that 254 nm is intrinsically more effective than nearby UV–C wavelengths should be made with caution, particularly when comparing different irradiation technologies operating under equivalent accumulated doses.

Overall, the present results demonstrate that accumulated UV–C dose remains the most appropriate parameter for comparing different UV–C disinfection technologies. Under rigorously controlled radiometric conditions, the low-pressure Hg lamp and the InGaN UV–C LED exhibited closely comparable dose–response behavior, kinetic parameters and characteristic inactivation doses. These findings suggest that, for surface disinfection of *S. aureus*, modern InGaN UV–C LEDs can provide germicidal performance comparable to that of conventional low-pressure mercury lamps while offering the well-known technological advantages associated with mercury-free operation, compactness, instant switching and increasing optical efficiency.

## Conclusions

This study presented a rigorous experimental comparison between a conventional low-pressure Hg lamp (254 nm) and an InGaN UV–C LED array (280 nm) under equivalent accumulated UV–C doses established through comprehensive spectral and radiometric characterization. By experimentally matching the irradiance at the sample plane before microbiological testing, dosimetric bias was minimized, enabling a controlled comparison between the two irradiation technologies.

Both irradiation systems produced rapid and effective inactivation of *S. aureus* ATCC 25923, exhibiting closely comparable dose–response behavior throughout the investigated dose range. A single-exponential model with a residual offset adequately described the experimental data and yielded model-estimated characteristic doses of similar magnitude for both technologies. The experimental survivor curves also agreed in order of magnitude with historical and contemporary susceptibility data, despite differences among the respective experimental protocols.

These results demonstrate that accumulated UV–C dose provides the most appropriate basis for comparing the two irradiation technologies and that comparisons based solely on nominal wavelength or exposure time may be misleading when radiometric equivalence is not ensured. Although 254 nm irradiation is traditionally regarded as the reference wavelength for UVGI because of its proximity to the nucleic-acid absorption maximum, the present results indicate that the evaluated 280 nm InGaN UV–C LED array achieved comparable germicidal performance when equivalent UV–C doses were delivered under controlled conditions. This comparability concerns dose-normalized microbial inactivation and should not be interpreted as equivalence in electrical efficiency.

Beyond demonstrating the potential of mercury-free UV–C LEDs for surface disinfection, this work establishes a reproducible experimental framework for the radiometrically equivalent evaluation of emerging UV–C technologies, providing a robust basis for future comparisons involving different wavelengths, microorganisms, and irradiation systems.

## Data Availability

All data supporting the findings of this study are provided within the article. Additional raw data (including original instrument outputs and analysis files) are available from the corresponding author upon reasonable request.

## Abbreviations

ATCC: American Type Culture Collection
CFU: colony-forming unit
DC: direct current
DNA: DNA
*D*_90_: UV–C dose required for 90% microbial inactivation
*D*_99_: UV–C dose required for 99% microbial inactivation
*D*_99.9_: UV–C dose required for 99.9% microbial inactivation
FWHM: full width at half-maximum
Hg: mercury
InGaN: indium gallium nitride
LED: light-emitting diode
MDF: medium-density fiberboard
UV: ultraviolet
UV-AOP: ultraviolet-based advanced oxidation process
UV–C: ultraviolet C
UVGI: ultraviolet germicidal irradiation
UV–vis: ultraviolet–visible
